# Effect of herbicides on soil respiration: a case study conducted at Debrecen-Látókép Plant Cultivation Experimental Station

**DOI:** 10.12688/f1000research.27057.1

**Published:** 2020-11-19

**Authors:** Zsolt Sándor, Ida Kincses, Magdolna Tállai, Daniel A. Lowy, Jesus R. Melendez, Nelly Ivonne Guananga Diaz, Luis Elias Guevara Iñiguez, Gerardo Cuenca Nevarez, Viviana Talledo Solórzano, János Kátai

**Affiliations:** 1 Institute of Agrochemistry and Soil Science, University of Debrecen, Debrecen, Hungary; 2 Research Group of Applied Plant Glycobiology, Dama Research Center limited, Kowloon, Hong Kong; 3 Genesis Sustainable Future, Ltd., Sárospatak, Hungary; 4 Facultad Educación Técnica para el Desarrollo, Universidad Católica de Santiago de Guayaquil, Guayaquil, Ecuador; 5 Escuela Superior Politécnica de Chimborazo, Riobamba, Ecuador; 6 Facultad de Ciencias Zootécnicas, Universidad Técnica de Manabí, Portoviejo, Ecuador

**Keywords:** CO2 emission, Chernozem, Herbicides, Látókép, Debrecen, soil respiration

## Abstract

Measuring the effect of herbicides on the natural environment is essential to secure sustainable agriculture practices. Amount of carbon dioxide released by soil microorganisms (soil respiration) is one of the most important soil health indicators, known so far. In this paper we present a comprehensive quantifying study, in which we measured the effect of 14 herbicides on soil respiration over 16 years, from 1991 to 2017, at Debrecen-Látókép Plant Cultivation Experimental Station. Investigated herbicides contained different active ingredients and were applied in various doses. It was found that 11 out of the examined 14 herbicides had a detrimental effect on soil respiration.

## Introduction

Carbon dioxide (CO _2_) is an important greenhouse gas, which affects significantly global warming and climate change ( Rastogi *et al*., 2002). Approximately 30% of the total CO _2 _emissions are released by agricultural activities. It is notable that agricultural CO _2_ emissions increased by 27% over two decades, from 1970 to 1990 ( Lal, 2004).

Primary sources of soil CO _2_ emissions are root respiration and degrading of organics by soil microorganisms. Soil microbial activity mainly depends on soil properties, including soil temperature, organic matter and soil moisture content ( Smith *et al*., 2003). Increasing scientific attention is focused on understanding the role of the soil microbial community ( Bautista *et al*., 2017; Cho-Tiedje, 2000; Mátyás *et al*., 2018; Mátyás *et al*., 2020) and nutrient cycles ( Jakab, 2020; Sándor *et al*., 2020). It has been documented that different cultivation technologies significantly impact soil microbiological activity ( Sándor *et al*., 2020).

Different chemicals (such as fertilizers and/or herbicides) are utilized in agricultural technologies. Use of herbicides constitutes an integral part of crop production, and one should be aware that they cause a “secondary effect” on both soil life and so called “non-target” organisms ( Kecskés, 1976). Sensitive organisms are killed after using herbicides, and their remains are easily decomposed by the surviving microorganisms ( Cervelli *et al*., 1978). At present, the selection criteria for allowed chemicals is more rigorous and stricter than over past decades, and they are restricted to smaller concentrations ( Inui *et al*., 2001). Soil microbes play a major role in maintaining soil quality ( Mendes *et al*., 2018; Wang *et al*., 2008).

In this paper, we discuss carbon dioxide emission levels of chernozem soil at the Debrecen-Látókép Plant Cultivation Experimental Station, where herbicides were applied to control the weeds. We compare results of carbon dioxide production in treated plots to untreated control parcels.

## Methods

First, we conducted a literature review on types and doses (L-ha ^-1^ or kg-ha ^-1^) of herbicides ( Molnár & Ocskó, 2000; Ocskó, 1991; Ocskó *et al*., 2017) that had been applied from 1991 to 2017 at Debrecen-Látókép Plant Cultivation Experimental Station (47°33’ 55.36” N; 21°28’ 12.27” E). The type of soil is calcareous chernozem; according to the International Classification (WRB) it is designated as Calcic Endofluvic Chernozen (Endosceletic). Prior absolute control soil was measured; control soil did not receive any treatment or fertilizers.

Soil CO _2_ was measured in triplicate by NaOH absorption. Experiments were performed between 1991 and 2017. In 1991, 2000, 2008 and 2017 soil samples were obtained two weeks after the herbicide(s) was applied. For incubation, 10 g of soil was weighed and placed in a polyester bag (0.1 mm ø holes), from where CO _2_ could escape. One took 500 mL laboratory glassware in which 10 cm ^3^ of 0.1 M NaOH solution (Sigma-Aldrich, USA) was introduced to absorb the released carbon dioxide. Soil samples were hung above the NaOH solution, and the glass containers were sealed tightly. Since CO _2_ has a greater density than air, it sunk in the container, and was absorbed by the alkaline solution. After an incubation period of 7 days, the remaining alkali solution was back titrated with 0.1 M HCl (Sigma-Aldrich, USA), in the presence of phenolphthalein, and then with methyl orange indicator. From the volume of equivalence one can calculate the amount of CO _2 _formed during soil respiration, according to Equation 1.

mg (CO _2_)· 10 g ^−1^ · 7 day ^−1^ = (C-S) · f(NaOH) · f(HCl) · 2.2 * dm            (1)

where, C: 0.1 M/ dm ^3^ HCl loss for methyl orange indicator (Sigma-Aldrich, USA); S: 0.1 M dm ^3^ HCl loss for phenolphthalein indicator (Sigma-Aldrich, USA); f: 0.1 mol dm ^-3^ HCl and a 0.10 mol dm ^-3^ NaOH factor; 2.2: titer (1 mL 0,1 mol dm ^-3^ HCl equivalent 2.2 mg CO _2_); dm: multiplication factor for dry soil.

Data analysis was performed using Microsoft Excel 2003 (mean values and standard deviation). Two-factor variance analysis was performed to obtain the significant effect on measured parameters. Significant differences were accepted at the level 1%, but the evaluation was calculated by LSD 5% values, as widely accepted in agricultural research.

## Results and discussion

In 1991, three herbicides were applied, and even the basic doses were high. Results were compared to the control; CO _2_ production was significantly reduced at single doses and a further decrease was experienced at 2–3 times greater doses. Consequently, CO _2_ production declined gradually with increasing doses of herbicides. The smallest production was obtained at 3 times the dose of Anelda Plus 80 EC, its value being only 59% of the control ( Table 1).

**Table 1.** Herbicides’ doses and soil respiration measured.

	Herbicide dose	Initial herbicide dose (L ha ^-1^)	1x	2x	3x
Year	Herbicide		Soil respiration (mg CO _2_ · 10 g ^-1^ · (7 day) ^-1^)		
1991	Control	None	23.5		
	Alirox 80 Ec	5-8	22.81	21.75	18.37
	Anelda Plus 80 EC	5-9	20.15	16.25	13.91
	Vernolate 80 EC	6-8	18.34	17.55	15.91
2000	Control	None	14.25		
	Dual 720 EC	2.5 -3.5	11.34	12.67	11.56
	Frontier 900 EC	1.5-2.0	10.4	10.21	10.14
	Hungazin PK	1.4-2.8 *	12.43	10.11	9.36
	Dual Gold 960 EC	1.4-1.6	10.52	10.37	10.21
	Proponit 8720 EC	1.5-2.5	12.4	11.71	10.54
	Acenit 880 EC	2.0-2.6	9.22	9.57	9.17
2008	Control	None	15.47		
	Merlin SC	0.16-0.20	15.41	15.38	15.49
	Wig EC	3.5-4.5	12.86	13.27	11.46
2017	Control	None	19.18		
	Adengo	0.40-0.44	19.42	19.47	19.44
	Capreno	0.25-0.30	18.87	19.16	18.96
	Figaro TF	2.0-5.0	17.94	17.72	16.3

* kg ha ^-1^ (quantity given in different units)

In 2000, six different active ingredients were used, and their effect examined. Much lower doses were applied, half and one third of the ones used in 1991. As compared to the control, soil respiration decreased significantly in all treated plots, after laboratory incubation. The lowest results were obtained with Acenit 880 EC; when 3x dose was used, only the 64% of the control being achieved.

In 2008, a significant decrease was found for the treated soil relative to the control. In the treatment with triple dose, only 74% of the control was measured. The herbicides used in 2008 are no longer authorized, as they were withdrawn from the market.

In 2017, three herbicides were examined. Out of them, Figaro TF, which contained glyphosate agent, was no longer authorized. When this herbicide was applied, CO _2_ production decreased significantly. Carbon dioxide production did not change considerably in Andengo and Capreno treatments; there was a slight increase in treatments with Andengo and decrease in treatments with Capreno.

## Conclusions

We can conclude that CO _2_ production decreased significantly in the soil for 11 out of the 14 herbicides. With two herbicides, Merlin SC (izoxaflutol) and Capreno (Isoxadifen-ethyl, tembotrione), there was no significant change of treated soils relative to the untreated soil, and there was only one herbicide Adengo (Bayer, Germany), which increased soil respiration slightly, but not significantly. The main sources of CO _2_-emissions from soil is the respiration of plant roots and of the microbial community. Therefore, a significant decrease of CO _2_ emission indicates a change in these parameters. One can recommend for use those chemicals, which do not cause major changes in the microbial community and do not affect life conditions of other live organisms. 

## Data availability

### Underlying data

Figshare: Supporting data CO2 soil respiration, https://doi.org/10.6084/m9.figshare.13125290.v1 ( Dama Research Center Limited, 2020). 

Data are available under the terms of the Creative Commons Attribution 4.0 International license (CC-BY 4.0). 
