# Supplementary material for: Effect of herbicides on soil respiration: a case study conducted at Debrecen-Látókép Plant Cultivation Experimental Station
Source: F1000Res. 2020 Nov 19;9:1348. [Version 1] doi: 10.12688/f1000research.27057.1 (PMC7919601; doi:10.12688/f1000research.27057.1)
Supplement: Peer review history

# Peer review: Reviewer response for version 1

**DOI:** 10.5256/f1000research.29886.r76811

**Published:** 2021-02-26

**Authors:** Istvan Fekete

**Affiliations:** 1 Institute of Environmental Sciences, University of Nyíregyháza, Nyíregyháza, Hungary

"Primary sources of soil CO _2_ emissions are root respiration and degrading of organics by soil microorganisms."

-  the above sentence needs some citatioms: e.g  Béni et al. 2017
  Beni Áron; Kate, Lajtha ; János, Kozma ; István, Fekete, (2017), Application of a Stir Bar Sorptive Extraction sample preparation method with HPLC for soil fungal biomass determination in soils from a detrital manipulation study, JOURNAL OF MICROBIOLOGICAL METHODS 136 pp. 1-5. ^1^
  PMID: **28238755, DOI: 10.1016/j.mimet.2017.02.009** 

"Soil microbial activity mainly depends on soil properties, including soil temperature, organic matter and soil moisture content Smith *et al*., 2003"

- A more recent citation would also be helpful.
  e. g. Fekete et al. 2021
  (Fekete, I ; Berki, I ; Lajtha, K ; Trumbore, S ; Francioso, O ; Gioacchini, P ; Montecchio, D ; Várbíró, G ; Béni, Á ; Makádi, M et al. (2021) How will a drier climate change carbon sequestration in soils of the deciduous forests of Central Europe?, BIOGEOCHEMISTRY 152 pp. 13-32. , 20 p.), https://doi.org/10.1007/s10533-020-00740-0 ^2^
  Kotroczó, Zsolt ; Koncz, Gábor ; Halász, L Judit ; Fekete, István ; Krakomperger, Zsolt ; Tóth, D Márta ; Balázsy, Sándor ; Tóth, János Attila, (2009) Litter decomposition intensity and soil organic matter accumulation in síkfőkút dirt site, ACTA MICROBIOLOGICA ET IMMUNOLOGICA HUNGARICA 56 pp. 53-54. , 2 p., DOI: 10.1556/AMicr.56.2009.Suppl.1 ^3^

How many repetitions did the measurements take, when the soil respiration values in Table 1 were determined? If average values were used then standard error or standard deviation values should also be included.

 It would also be useful to indicate in the table which values differed significantly from the control.

Is the work clearly and accurately presented and does it cite the current literature?

Partly

If applicable, is the statistical analysis and its interpretation appropriate?

Partly

Are all the source data underlying the results available to ensure full reproducibility?

Partly

Is the study design appropriate and is the work technically sound?

No

Are the conclusions drawn adequately supported by the results?

Yes

Are sufficient details of methods and analysis provided to allow replication by others?

Yes

Reviewer Expertise:

Soil ecology

I confirm that I have read this submission and believe that I have an appropriate level of expertise to confirm that it is of an acceptable scientific standard.

## References

[1] : Application of a Stir Bar Sorptive Extraction sample preparation method with HPLC for soil fungal biomass determination in soils from a detrital manipulation study. *J Microbiol Methods*.136: 10.1016/j.mimet.2017.02.009 1-5 10.1016/j.mimet.2017.02.009 28238755

[2] : Correction to: How will a drier climate change carbon sequestration in soils of the deciduous forests of Central Europe?. *Biogeochemistry*.2021;152(1) : 10.1007/s10533-020-00740-0 33-34 10.1007/s10533-020-00740-0

[3] : Litter decomposition intensity and soil organic matter accumulation in síkfőkút dirt site. *ACTA MICROBIOLOGICA ET IMMUNOLOGICA HUNGARICA*.2009; Reference source

# Peer review: Reviewer response for version 1

**DOI:** 10.5256/f1000research.29886.r75106

**Published:** 2021-01-07

**Authors:** Fa-Jun Chen

**Affiliations:** 1 Department of Entomology, Nanjing Agricultural University, Nanjing, China

In this manuscript, the authors carried out experiments to study the effect of herbicides on soil respiration. In this study, they measured the effect of 14 herbicides on soil respiration over 16 years, from 1991 to 2017, at Debrecen-Látókép Plant Cultivation Experimental Station.This is an interesting and novel topics for the readers.

 There were some errors and shortcomings, which were following as:

-  Q1: Title: In this title, it is no necessary to give the testing site. So it should be changed as “Effect of herbicides on soil respiration: a case study”.
- Q2: Abstract: Give more results of the measured indexes, not just the description of detrimental effects!
- Q3: M&M: Soil CO2 was measured in triplicate by NaOH absorption. Experiments were performed between 1991 and 2017. Why was just soil samples were obtained two weeks after the herbicide(s) in 1991, 2000, 2008 and 2017? What about the results of the other measuring years?  And what were the two factors in the two-favtor ANOVAs? Sampling years and herbicide treatment? This should be given in the data analysis.
- Q4: Results: Why were just the results in 1991, 2000, 2008 and 2017 given in this study? What about the other years' results from 1991 to 2017?
- Q5: Table 1: In this stable, it shows confused and not clear to say "Herbicide dose", "Initial herbicide dose" (L ha-1), and 1x, 2x and3x!

Is the work clearly and accurately presented and does it cite the current literature?

Yes

If applicable, is the statistical analysis and its interpretation appropriate?

Yes

Are all the source data underlying the results available to ensure full reproducibility?

Partly

Is the study design appropriate and is the work technically sound?

Partly

Are the conclusions drawn adequately supported by the results?

Yes

Are sufficient details of methods and analysis provided to allow replication by others?

Yes

Reviewer Expertise:

Plant Protection; Insect Ecology; Soil Ecology

I confirm that I have read this submission and believe that I have an appropriate level of expertise to confirm that it is of an acceptable scientific standard, however I have significant reservations, as outlined above.

# Peer review: Reviewer response for version 1

**DOI:** 10.5256/f1000research.29886.r75105

**Published:** 2020-12-01

**Authors:** Alaa El-Dien Omara, Sahar El-Nahrawy

**Affiliations:** 1 Department of Agricultural Microbiology, Soil,Water and Environment Research Institute, Agricultural Research Center (ARC), Giza, Egypt; 2 Department of Agricultural Microbiology, Soil,Water and Environment Research Institute, Agricultural Research Center, Giza, Egypt

The authors provided greater clarity on the controls for their study, which measured the effect of 14 herbicides on soil respiration over 16 years from 1991 to 2017, and this study is highly interesting and readers will be intrigued by these results.

 I have some minor comment that are:

- Creating a map with the sites for sampling to be studied with a mention of the crops grown during the study period;
- The number of doses should be indicated in the methods used;
- What about the standard deviation and LSD in Table 1.

In the future, I would like to use a phospholipid fatty acids (PLFAs) technique to study the biomass of the microbial community such as bacteria, fungi….etc.

Is the work clearly and accurately presented and does it cite the current literature?

Yes

If applicable, is the statistical analysis and its interpretation appropriate?

Partly

Are all the source data underlying the results available to ensure full reproducibility?

Yes

Is the study design appropriate and is the work technically sound?

Yes

Are the conclusions drawn adequately supported by the results?

Yes

Are sufficient details of methods and analysis provided to allow replication by others?

Yes

Reviewer Expertise:

Soil microbiology

We confirm that we have read this submission and believe that we have an appropriate level of expertise to confirm that it is of an acceptable scientific standard.
